# Lipid goal attainment in diabetes mellitus patients after acute coronary syndrome: a subanalysis of Dyslipidemia International Study II-China

**DOI:** 10.1186/s12872-023-03312-w

**Published:** 2023-07-01

**Authors:** Tongshuai Guo, Chao Chu, Yang Wang, Mingjun He, Hao Jia, Yue Sun, Dan Wang, Yan Liu, Yong Huo, Jianjun Mu

**Affiliations:** 1 Department of Cardiovascular Medicine, First Affiliated Hospital of Xi’an Jiaotong University, No. 277 Yanta West Road, 710061 Xi’an, China; 2 Medical Affairs, Organon, China; 3 Department of Cardiovascular Medicine, Peking University First Hospital, Beijing, China; 4 Department of Cardiology, Peking University First Hospital, No. 8 Xishiku Street, Xicheng District, 100034 Beijing, China

**Keywords:** Low-density lipoprotein cholesterol goal, Acute coronary syndrome, Diabetes mellitus, Lipid-lowing therapy, Statin

## Abstract

**Background:**

Lipid management with a low-density lipoprotein cholesterol (LDL-C) goal of < 1.4 mmol/L is recommended for patients with acute coronary syndrome (ACS) and diabetes mellitus (DM) due to a high risk for adverse cardiovascular events. This study evaluated the lipid-lowering treatment (LLT) pattern and the LDL-C goal attainment rate in this special population.

**Methods:**

DM patients were screened from the observational Dyslipidemia International Study II-China study which assessed LDL-C goal attainment in Chinese ACS patients. The baseline characteristics between the LLT and no pre-LLT groups were compared. The proportions of patients obtaining LDL-C goal at admission and at 6-months, the difference from the goal, and the pattern of the LLT regimen were analyzed.

**Results:**

Totally 252 eligible patients were included, with 28.6% taking LLT at admission. Patients in the LLT group were older, had a lower percentage of myocardial infarction, and had decreased levels of LDL-C and total cholesterol compared to those in the no pre-LLT group at baseline. The overall LDL-C goal attainment rate was 7.5% at admission and increased to 30.2% at 6 months. The mean difference between the actual LDL-C value and LDL-C goal value dropped from 1.27 mmol/L at baseline to 0.80 mmol/L at 6 months. At 6 months, 91.4% of the patients received statin monotherapy, and only 6.9% received a combination of statin and ezetimibe. The atorvastatin-equivalent daily statin dosage was moderate during the study period.

**Conclusion:**

The low rate of lipid goal attainment observed was in line with the outcomes of other DYSIS-China studies.

**Supplementary Information:**

The online version contains supplementary material available at 10.1186/s12872-023-03312-w.

## Background

Cardiovascular disease (CVD) is the leading cause of death worldwide, in which a large proportion of patients have acute coronary syndrome (ACS) and stable coronary artery disease [1]. Diabetes mellitus (DM) is a chronic metabolic disease that is highly prevalent in patients with CVD [2, 3]. In the setting of ACS, the presence of DM is a robust independent predictor of recurrent ischemic events and hence a dismal patient prognosis even after ACS treatment [4, 5]. Lifestyle modification and early, intensive lipid-lowering therapy (LLT) are recommended to reduce recurrent events in patients with ACS [4, 6]. For those with preexisting DM who belong to the very high-risk population, extremely strict low-density lipoprotein cholesterol (LDL-C) management is required to reach a target goal of 1.4 mmol/L LDL-C, which is based on the latest guidelines of the European Society of Cardiology (ESC) and European Atherosclerosis Society (EAS) as well as the consensus of the Chinese College of Emergency Physicians (CCEP) [7, 8]. However, there are insufficient data regarding the real-world LDL-C goal attainment rate in ACS patients with DM in China.

The nationwide Dyslipidemia International Study-China (DYSIS-China) study enrolling 25,697 patients receiving LLT showed that the percentages of high-risk and very-high-risk patients who achieved the goal were only 39.7% [9]. Further analyses based on the global DYSIS II study demonstrated that only 11.3% of type 2 DM patients hospitalized for ACS achieved the LDL-C goal of < 1.4 mmol/L at admission, and this attainment rate was improved to 22.5% at the 4-month follow-up [10]. For Chinese patients, the DYSIS II-China study aimed to assess the LDL-C goal (< 1.8 mmol/L) attainment rate in post-ACS patients including DM patients, who accounted for 28.6% of the whole population [11]. Hence, in the present analysis of the DYSIS II-China study, we attempted to specifically investigate the LLT patterns and LDL-C goal attainment of < 1.4 mmol/L at baseline and at 6 months post-ACS in DM patients.

## Methods

### Patients

The medical records of the DM patients enrolled in this study were extracted from the DYSIS II-China study database. The DYSIS II-China study was a multicenter prospective observational study conducted between September 2017 and May 2019, which aimed to assess LDL-C goal attainment in Chinese patients with ACS [11]. The inclusion criteria were as follows: (1) age ≥ 18 years old, (2) written informed consent was obtained, (3) hospitalized for ACS, (4) complete lipid profile within 24 h after admission, (5) on LLT for ≥ 3 months at the time of enrollment, or not at all on LLT. Patients with LLT for < 3 months, who died, or who had any cognitive impairment at discharge were excluded. DM was defined as current treatment for diabetes, a previous diagnosis of diabetes or a fasting plasma glucose concentration ≥ 126 mg/dL [12].

The DYSIS II-China study was carried out in accordance with the Declaration of Helsinki. All participating patients provided written informed consent. The protocol for this study was approved by the Ethics Committee of Peking University First Hospital, according to the local regulations.

### Data collection

The demographic, clinical, and laboratory variables were collected from the database. The demographic and clinical data included age, sex, body mass index, type of ACS, sedentary lifestyle, smoking status, and comorbidities. The laboratory data included baseline high-sensitivity C-reactive protein (hsCRP), fasting plasma glucose, and glycated hemoglobin A1c (HbA1c) levels as well as the lipid profile (serum levels of total cholesterol [6], LDL-C, high-density lipoprotein cholesterol (HDL-C), non-HDL-C, and triglycerides) at baseline and at the 6-month follow-up visit. Lipid control was defined as achieving the goal of LDL-C < 1.4 mmol/L, according to the 2019 ESC guidelines and 2019 CCEP consensus [8, 13].

### Statistical analysis

Continuous variables were expressed as the mean ± standard deviation or median and interquartile range, as appropriate. Categorical variables were reported as a number and percentage. The chi-squared test, T test, and paired-sample T test were used to compare the differences between groups. Multivariate logistic regression analysis was performed for identifying the predictors of LDL-C goal attainment. Analyses were performed using SPSS 23.0 software. A two-sided *P-*value < 0.05 was considered statistically significant.

## Results

### Patient disposition

**Fig. 1:**
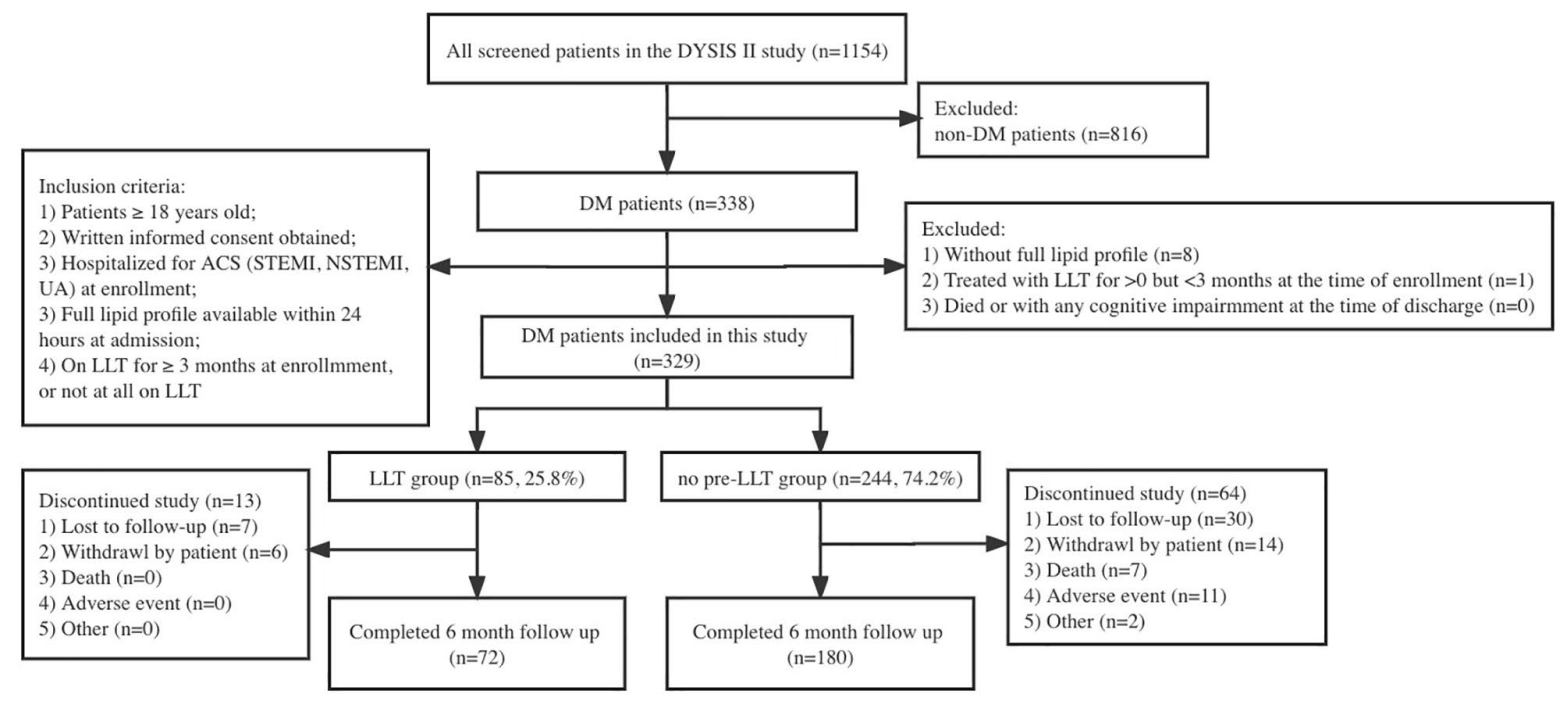
Flow chart of this study. Abbreviations: ACS, acute coronary syndrome; ICF, informed consent form; LLT, lipid-lowering therapy; NSTEMI, non-ST elevation myocardial infarction; STEMI, ST elevation myocardial infarction; UA, unstable angina. LLT group: Patients on LLT ≥ 3 months; no pre-LLT group: LLT-naive or off LLT for ≥ 3 months

All patients (*n* = 1154) in the DYSIS II-China database were screened. In total, 338 patients with DM were initially enrolled. Among whom, 252 patients with DM were eventually included in this study. There were 72 patients in the LLT group and 180 in the no pre-LLT group who returned for the 6-month clinic visits and completed the study assessments (Fig. 1).

### Baseline demographics and clinical profile

The average age of the study population was 63.2 ± 10.5 years old, and 74.2% of the patients were men. In terms of the ACS type, unstable angina (57.5%) was more prevalent than ST elevation myocardial infarction (STEMI; 26.2%) and non-ST elevation myocardial infarction (NSTEMI; 16.3%). Smokers accounted for 42.9% of the population, and 30.6% of the patients had a sedentary lifestyle. The median hsCRP concentration was 3.8 mg/L. Among 240 patients with a recorded DM type, only one patient (0.4%) was diagnosed with type 1 DM, and all others had type 2 DM. The mean fasting plasma glucose level was 8.6 ± 3.2 mmol/L, and the mean HbA1c level was 7.9 ± 1.6% (Table 1).

**Table 1:** Demographic and clinical profile

Characteristic/category	All patients (*n* = 252)	No pre-LLT patients (*n* = 180)	LLT patients (*n* = 72)	*P*-value*
**Age**, mean ± SD (years)	63.2 ± 10.5	61.8 ± 10.7	66.5 ± 9.3	**0.002**
**Sex**				0.649
Male	187 (74.2%)	135 (75.0%)	52 (72.2%)	
Female	65 (25.8%)	45 (25.0%)	20 (27.8%)	
**Type of ACS**				**0.026**
STEMI	66 (26.2%)	53 (29.4%)	13 (18.1%)	
NSTEMI	41 (16.3%)	33 (18.3%)	8 (11.1%)	
UA	145 (57.5%)	94 (52.2%)	51 (70.8%)	
**Cigarette smoking status**				0.831
Never	142 (56.3%)	102 (56.7%)	40 (55.6%)	
Current	77 (30.6%)	54 (30.0%)	23 (31.9%)	
Former	31 (12.3%)	22 (12.2%)	9 (12.5%)	
Unknown	2 (0.8%)	2 (1.1%)	0 (0.0%)	
**DM**				0.213
Previously diagnosed	229 (90.9%)	161 (89.4%)	68 (94.4%)	
Newly diagnosed	23 (9.1%)	19 (10.6%)	4 (5.6%)	
**Sedentary lifestyle**				0.901
Yes	77 (30.6%)	55 (30.6%)	22 (30.6%)	
No	147 (58.3%)	104 (57.8%)	43 (59.7%)	
Unknown	28 (11.1%)	21 (11.7%)	7 (9.7%)	
**hsCRP**, median (range), mg/L	3.8 (0.0-178.0)	4.1 (0.0-178.0)	2.0 (0.1-112.3)	0.077
**Fasting plasma glucose**, mmol/L	8.6 ± 3.2	8.8 ± 3.4	8.3 ± 2.9	0.423
**HbA1c**, %	7.9 ± 1.6	7.9 ± 1.7	7.7 ± 1.5	0.429
**BMI**, mean ± SD (kg/m^2^)	25.5 ± 3.3	25.4 ± 3.4	25.8 ± 3.2	0.541
**History of previous ACS**				0.235
Yes	91 (36.1%)	68 (37.8%)	23 (31.9%)	
No	146 (57.9%)	104 (57.8%)	42 (58.3%)	
Unknown	15 (6.0%)	8 (4.4%)	7 (9.7%)	
**History of previous MI**				0.563
Yes	44 (17.5%)	34 (18.9%)	10 (13.9%)	
No	194 (77.0%)	137 (76.1%)	57 (79.2%)	
Unknown	14 (5.6%)	9 (5.0%)	5 (6.9%)	
**History of ischemic heart disease**				0.305
Yes	46 (18.3%)	30 (16.7%)	16 (22.2%)	
No	194 (77.0%)	143 (79.4%)	51 (70.8%)	
Unknown	12 (4.8%)	7 (3.9%)	5 (6.9%)	
**Prior angina**				0.091
Yes	86 (34.1%)	63 (35.0%)	23 (31.9%)	
No	151 (59.9%)	110 (61.1%)	41 (56.9%)	
Unknown	15 (6.0%)	7 (3.9%)	8 (11.1%)	
**History of coronary revascularization**				0.656
Yes	63 (25.0%)	44 (24.4%)	19 (26.4%)	
No	179 (71.0%)	130 (72.2%)	49 (68.1%)	
Unknown	10 (4.0%)	6 (3.3%)	4 (5.6%)	
**History of TIA**				0.242
Yes	5 (2.0%)	2 (1.1%)	3 (4.2%)	
No	231 (91.7%)	167 (92.8%)	64 (88.9%)	
Unknown	16 (6.3%)	11 (6.1%)	5 (6.9%)	
**History of intermittent claudication**				0.596
Yes	1 (0.4%)	1 (0.6%)	0 (0.0%)	
No	232 (92.1%)	167 (92.8%)	65 (90.3%)	
Unknown	19 (7.5%)	12 (6.7%)	7 (9.7%)	
**History of peripheral artery revascularization**				0.716
Yes	3 (1.2%)	2 (1.1%)	1 (1.4%)	
No	235 (93.3%)	169 (93.9%)	66 (91.7%)	
Unknown	14 (5.6%)	9 (5.0%)	5 (6.9%)	
**History of symptomatic CHF**				0.149
Yes	24 (9.5%)	21 (11.7%)	3 (4.2%)	
No	215 (85.3%)	151 (83.9%)	64 (88.9%)	
Unknown	13 (5.2%)	8 (4.4%)	5 (6.9%)	
**Hypertension**				0.896
No	65 (25.8%)	46 (25.6%)	19 (26.4%)	
Yes, previously diagnosed	178 (70.6%)	128 (71.1%)	50 (69.4%)	
Yes, newly diagnosed	9 (3.6%)	6 (3.3%)	3 (4.2%)	
**History of CKD**				0.687
No	228 (90.5%)	162 (90.0%)	66 (91.7%)	
Yes, previously diagnosed	8 (3.2%)	7 (3.9%)	1 (1.4%)	
Yes, newly diagnosed	2 (0.8%)	1 (0.6%)	1 (1.4%)	
Unknown	14 (5.6%)	10 (5.6%)	4 (5.6%)	
**Hypercholesterolemia**				0.970
No	200 (79.4%)	142 (78.9%)	58 (80.6%)	
Yes, previously diagnosed	26 (10.3%)	19 (10.6%)	7 (9.7%)	
Yes, newly diagnosed	6 (2.4%)	4 (2.2%)	2 (2.8%)	
Unknown	20 (7.9%)	15 (8.3%)	5 (6.9%)	
**History of stroke**				0.368
Yes	20 (7.9%)	17 (9.4%)	3 (4.2%)	
No	214 (84.9%)	150 (83.3%)	64 (88.9%)	
Unknown	18 (7.1%)	13 (7.2%)	5 (6.9%)	
**Chronic lung disease**				0.829
Yes	10 (4.0%)	8 (4.4%)	2 (2.8%)	
No	228 (90.5%)	162 (90.0%)	66 (91.7%)	
Unknown	14 (5.6%)	10 (5.6%)	4 (5.6%)	

LLT, lipid-lowering therapy; SD, standard deviation; ACS, acute coronary syndrome; STEMI, ST elevation myocardial infarction; NSTEMI, non-ST elevation myocardial infarction; UA, unstable angina; DM, Diabetes mellitus; hsCRP, high-sensitivity C-reactive protein; HbA1c: glycated hemoglobin A1c; BMI, body mass index; MI, myocardial infarction; TIA: transient ischemic attack; CHF, chronic heart failure; CKD, chronic kidney disease

*P-value compared between the no pre-LLT group and the LLT group

The baseline characteristics of the study population (n = 252) were compared to those who discontinued the study (n = 77). There were no significant differences, except that those who dropped out had a higher proportion of stroke history (Additional File Table 1).

At admission, 28.6% of the patients were on LLT. The patients in the LLT group were older and had a higher percentage of unstable angina compared to those in the no pre-LLT group (Table 1).

### Attainment of lipid goals

At admission, the LLT group had remarkably lower levels of total cholesterol (3.8 ± 1.0 vs. 4.3 ± 1.1 mmol/L, *P* < 0.001) and LDL-C (2.3 ± 0.8 vs. 2.7 ± 1.0 mmol/L, *P* = 0.002) compared to the no pre-LLT group. However, at the 6-month follow-up, there were no differences between the LLT and no pre-LLT groups in terms of all lipid parameters. The mean LDL-C level at the 6-month follow-up for all patients was less than that at baseline (2.6 ± 0.9 to 1.7 ± 1.0 mmol/L). While for the LLT and no pre-LLT groups, the levels of LDL-C at the 6-month follow-up were both 1.7 ± 1.0 mmol/L (Table 2).

**Table 2:** Lipid levels at admission and at the 6-month follow-up

Characteristic/category	All patients (*n* = 252)	No pre-LLT patients (*n* = 180)	LLT patients (*n* = 72)	*P*-value*
**Lipid levels at admission**, mean ± SD (mmol/L)				
TC	4.2 ± 1.1	4.3 ± 1.1	3.8 ± 1.0	**< 0.001**
LDL-C	2.6 ± 0.9	2.7 ± 1.0	2.3 ± 0.8	**0.002**
HDL-C	1.0 ± 0.3	1.0 ± 0.3	1.0 ± 0.2	0.141
TG	2.0 ± 1.8	2.1 ± 2.0	1.6 ± 0.9	0.051
**Lipid levels at 6-month follow-up**, mean ± SD (mmol/L)				
TC	3.1 ± 1.6	3.1 ± 1.5	3.1 ± 1.6	0.834
LDL-C	1.7 ± 1.0	1.7 ± 1.0	1.7 ± 1.0	0.481
HDL-C	0.9 ± 0.5	0.9 ± 0.5	0.9 ± 0.5	0.744
TG	1.5 ± 1.7	1.5 ± 1.9	1.3 ± 1.1	0.619
**LDL-C < 1.4 mmol/L at admission**				0.821
No	233 (92.5%)	166 (92.2%)	67 (93.1%)	
Yes	19 (7.5%)	14 (7.8%)	5 (6.9%)	
**LDL-C < 1.4 mmol/L at 6-month follow-up**				0.491
No	174 (69.0%)	122 (67.8%)	52 (72.2%)	
Yes	78 (31.0%)	56 (31.5%)	20 (27.8%)	

TC, total cholesterol; LDL-C, low-density lipoprotein cholesterol; HDL-C, high-density lipoprotein cholesterol; TG, triglycerides

*P-value compared between the no pre-LLT group and the LLT group

**Fig. 2:**
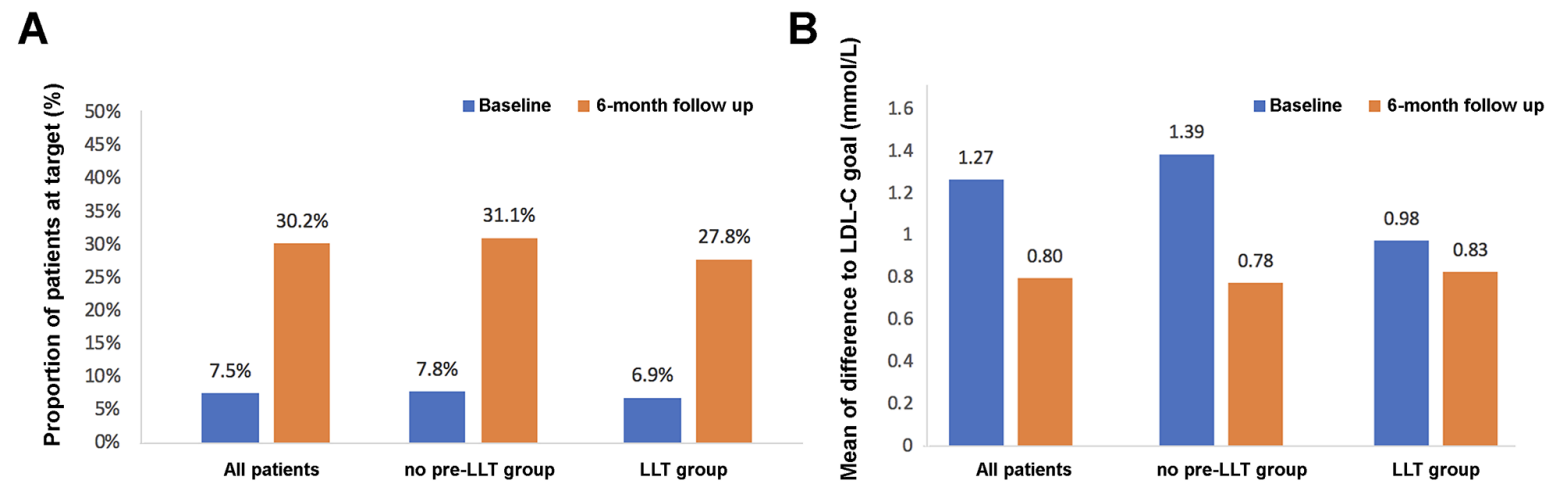
**(A)** LDL-C goal (< 1.4 mmol/L) attainment rate at baseline and the 6-month follow-up. **(B)** Difference between the actual LDL-C level and the LDL-C goal value at baseline and at the 6-month follow-up

At admission, the proportions of patients achieving the LDL-C goal (< 1.4 mmol/L) were only 7.5% for the overall population, 7.8% for the no pre-LLT group, and 6.9% for the LLT group. However, at the 6-month follow-up, a significantly higher percentage of patients from both the no pre-LLT group (31.1%) and LLT group (27.8%) had achieved the LDL-C goal (Fig. 2A).

In patients who did not reach the LDL-C goal, the mean difference to the LDL-C goal dropped from 1.27 mmol/L at baseline to 0.80 mmol/L at the 6-month follow-up. The corresponding differences were 1.39–0.78 mmol/L in the no pre-LLT group and 0.98–0.83 mmol/L in the LLT group (Fig. 2B).

Among all baseline characteristics, comorbidity hypertension was negatively predicted LDL-C goal attainment (Odds Ratio: 0.50, 95% Confidence Interval, 0.26 to 0.97, p = 0.041, Table 3).

**Table 3:** Predictors for attaining LDL-C < 1.4 mmol/L at 6-month follow-up

Characteristic	Odds Ratio (95% Confidence Interval	*P*-value
Age ≥ 65 years	1.53 (0.83–2.82)	0.178
BMI ≥ 28 kg/m2	0.95 (0.46–1.97)	0.885
Gender (male)	1.24 (0.61–2.48)	0.554
Type of ACS (STEMI)	0.46 (0.11–1.86)	0.274
Current smoker	1.16 (0.60–2.26)	0.653
Sedentary lifestyle	1.87 (0.99–3.54)	0.053
History of previous ACS	1.46 (0.62–3.43)	0.385
History of previous MI	0.72 (0.26–2.01)	0.528
History of ischemic heart disease	1.84 (0.76–4.45)	0.178
Prior angina	1.35 (0.60–3.01)	0.467
History of coronary revascularization	0.41 (0.15–1.09)	0.073
History of TIA	0.35 (0.03–4.12)	0.402
History of intermittent claudication	/	/
History of peripheral artery revascularization	1.86 (0.10-34.19)	0.677
History of symptomatic CHF	1.48 (0.54–4.08)	0.446
Hypertension	**0.50 (0.26–0.97)**	**0.041**
History of CKD	1.04 (0.26–4.18)	0.961
Hypercholesterolemia	0.47 (0.17–1.32)	0.151
History of stroke	2.32 (0.83–6.50)	0.109
Chronic lung disease	0.20 (0.02–1.78)	0.150
Further PCI during hospitalization	1.76 (0.39–7.92)	0.462

BMI, body mass index; STEMI, ST elevation myocardial infarction; ACS, acute coronary syndrome; MI, myocardial infarction; TIA: transient ischemic attack; CHF, chronic heart failure; CKD, chronic kidney disease; PCI, percutaneous coronary intervention

### LLT

**Fig. 3:**
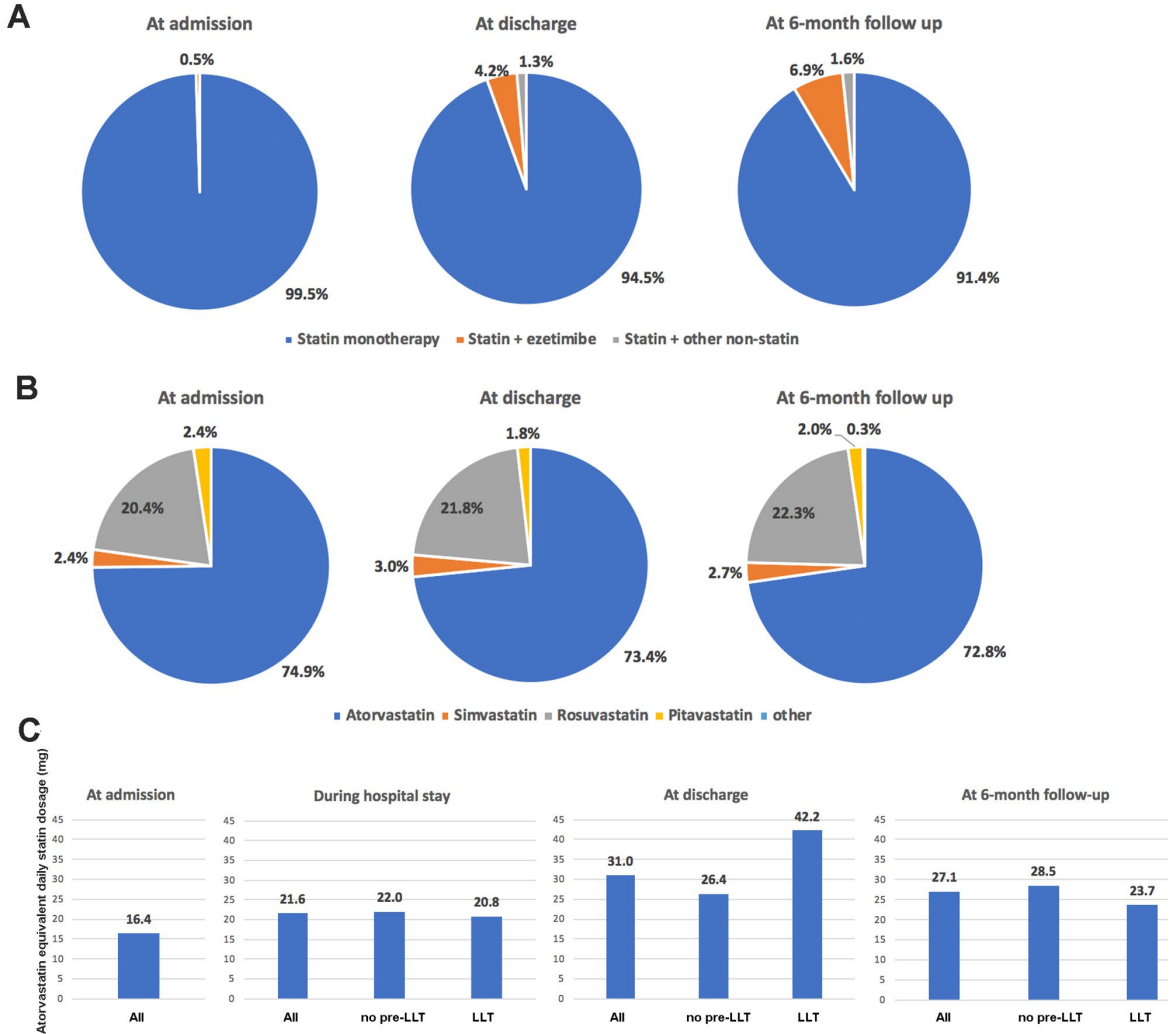
Lipid-lowering therapy (LLT). **(A)** The LLT treatment pattern in all DM patients at three timepoints: Admission, discharge, and 6-month follow-up; **(B)** Statin category in all DM patients at the three timepoints: Admission, discharge, and 6-month follow-up; **(C)** Atorvastatin-equivalent daily statin dosage at four timepoints in all patients, the LLT group, and the no pre-LLT group. Atorvastatin-equivalent dose calculation: atorvastatin (5 mg) = simvastatin (10 mg), fluvastatin (40 mg), lovastatin (20 mg), pravastatin (20 mg), pitavastatin (1 mg); atorvastatin (10 mg) = simvastatin (20 mg), fluvastatin (80 mg), lovastatin (40 mg), pravastatin (40 mg), pitavastatin (2–4 mg), rosuvastatin (5 mg); atorvastatin (20 mg) = simvastatin (40 mg), lovastatin (80 mg), pravastatin (80 mg), rosuvastatin (10 mg); atorvastatin 40 mg = simvastatin (80 mg), rosuvastatin (20 mg); atorvastatin (80 mg) = rosuvastatin (40 mg)

The most frequently used LLT was statin monotherapy, namely atorvastatin monotherapy, at all timepoints during the study (Fig. 3). The mean atorvastatin or atorvastatin-equivalent dose was 16.4 mg/day at admission (LLT group). For all patients, the atorvastatin-equivalent doses were 21.6 mg/day during the hospital stay, 31.0 mg/day at discharge, and 27.1 mg/day at the 6-month follow-up. For the LLT group, the doses were 20.8, 42.2, and 23.7 mg/day, respectively (Fig. 3).

At admission, only three patients (4.2%) in the LLT group were on an atorvastatin-equivalent dose of 40 mg/day, and no patient was on high-dose statin therapy (atorvastatin or atorvastatin-equivalent dose > 40 mg/day). Twenty-nine patients (11.5%) [LLT: 4/72 (5.6%), no pre-LLT: 25/180 (13.9%)] at discharge and 32 patients (12.7%) at the 6-month follow-up [LLT: 6/72 (8.3%), no pre-LLT: 26/180 (14.4%)] were prescribed with an atorvastatin-equivalent dose of 40 mg/day. In the no pre-LLT group, three patients (1.7%) at discharge and four patients (2.2%) at the 6-month follow-up were on high-dose statin therapy (atorvastatin-equivalent dose of > 40 mg/day).

## Discussion

ACS patients with DM are classified as being in a very-high risk group for adverse cardiovascular events [2]. The current study focusing on diabetic patients from the real-world DYSIS II-China study revealed an unsatisfactory level of lipid control for this subgroup. Merely 7.5% of the patients with DM had an LDL-C goal achievement of < 1.4 mmol/L at ACS onset, though this rate increased to 31% at the 6-month follow-up.

The included DM patients accounted for approximately one-fourth of all patients enrolled in the DYSIS II-China study. We observed a numerically lower LDL-C goal achievement rate for the DM subset compared with the overall DYSIS II-China population. For the overall Chinese ACS population, with the LDL-C goal cut-off set at < 1.8 mmol/L, 17.1% of the patients at admission and 41.2% of the patients at follow-up had achieved the LDL-C goal [11]. Similar to the baseline characteristics in the overall population, about three-fourths of the DM patients were male, and hypertension was the most common comorbidity [11]. In terms of the baseline characteristics between the DM patients with vs. without LLT, no significant disparities were observed except that the elderly patients were more likely to receive LLT at baseline and less occurrence of STEMI and NSTEMI was observed in the LLT group.

Moderate-intensity statin therapy is advised by the Chinese guidelines/expert consensus for LLT therapy based on efficiency and safety considerations [8, 14, 15]. Previous studies have demonstrated that high-intensity LLT may not produce greater cardiovascular benefits compared with medium-intensity therapy [16]. The CHILLAS trial in a Chinese ACS population has shown that doubling the statin dosage to 40 mg instead of 20 mg only resulted in an LDL-C reduction of 6.4% [16]. Other studies on adult Asian patients with dyslipidemia also have demonstrated that medium- and high-intensity statins lowered LDL-C levels by a similar proportion or similar proportions of patients who had achieved the LDL-C goals [17, 18]. However, it is noteworthy that the LDL-C target for most dyslipidemia patients is less stringent than that for DM patients post-ACS. As seen in the current study, the atorvastatin-equivalent daily statin dosage was moderate at 27 mg at the 6-month follow-up. Nevertheless, there still existed a large gap between the LDL-C goal recommended by guidelines and the real-world situation. Numerous studies have demonstrated a strong correlation between DM and hypertension [19, 20]. Our research found that concurrent hypertension was a negative factor for LDL-C goal achievement. This finding is in line with the result of the DYSIS-China study, which demonstrated that hypertensive dyslipidemia patients had a low rate of attaining their LDL-C goals [21]. Overall, less than one-third of the patients in our cohort finally reached the LDL-C goal, and there was no difference between the LLT group and no pre-LLT group, indicating a necessity for effective escalation of the LLT strategy.

The majority of patients in the current study used a single moderate-intensity statin agent, which is in line with the situation displayed in the DYSIS-China and DYSIS II-China studies [9, 11]. However, the rate of reaching the target lipid levels was not ideal in the DYSIS series studies. When moderate-intensity statin use cannot satisfy lipid-lowering needs, a statin combined with the cholesterol-absorption inhibitor ezetimibe is considered as the preferred intensive LLT regimen in China [22]. This combination further enhances the cholesterol-lowering efficacy with LDL-C reduction by more than 50% [22, 23]. However, we found that only 0.5% of the DM patients at baseline used satin plus ezetimibe, and this proportion increased to 6.9% at follow-up, thus indicating underutilization of combination treatments both initially and at follow-up.

The initial combination LLT therapy is feasible for very-high-risk patients such as DM patients who had ACS. In the IMPROVE-IT study, two-thirds of patients initially had combination therapy of statin and ezetimibe, and they demonstrated a long-term intensive reduction of LDL-C to 50 mg/dL, which further decreased their risk of cardiovascular events without significant safety concerns different from single-statin use [23]. More importantly, the benefit of adding ezetimibe to statin therapy was enhanced in patients with DM [24]. In addition, the FOURIER and ODYSSEY trials proved that proprotein convertase subtilisin/kexin 9 (PCSK9) inhibitors effectively reduce LDL-C levels and cardiovascular events [25, 26]. Besides their LDL-C lowering and positive prognostic impact, PCSK9 inhibitors have been shown to improve the quality of life of patients at high or very high cardiovascular risk [27]. Data from European practice indicated that PCSK9 inhibitors had a higher level of adherence than statins in high-risk patients, likely due to their favorable administration regimen and low rates of adverse effects [28]. However, considering the insufficient data on their long-term safety and unclear cost-effectiveness in China, their use may be limited for Chinese patients.

There are several limitations of this study that must be addressed. First, it is a secondary analysis based on the DYSIS II-China database. The results may not reflect the current situation. Second, the sample size was small, and 23% of the initially screened DM patients discontinued the study mainly due to loss at follow-up; hence, our results of LDL-C goal attainment might be overestimated. Third, selection bias may have existed because all participating centers were tertiary hospitals. Moreover, physician-related factors and medication adherence were not investigated in this study. Fourth, all reported adverse events were cardiovascular-related, and LLT especially statin-related adverse reaction were not investigated. Additionally, given the definition of DM used in this study, patients with only high postprandial glucose may have not been included in this DM population.

## Conclusions

In conclusion, moderate-intensity statin monotherapy was the predominant LLT for DM patients in the DYSIS II-China study. However, for this very-high-risk subset of patients, there was a low rate of lipid goal attainment at ACS occurrence and at 6 months after discharge. Therefore, intensified statin use and combination therapy are encouraged.

## Electronic supplementary material

Below is the link to the electronic supplementary material.

**Additional File Table 1:** Demographic, clinical profile and lipid levels at admission of patients included in the final analysis and those discontinued the study

## Data Availability

The datasets generated and analyzed during the current study are not publicly available due to limitations of ethical approval involving the patient data and anonymity but are available from the corresponding author on reasonable request.

## List of abbreviations

ACS: acute coronary syndrome
BMI: body mass index
CHF: chronic heart failure
CKD: chronic kidney disease
CVD: Cardiovascular disease
CCEP: Chinese College of Emergency Physicians
DM: diabetes mellitus
DYSIS: Dyslipidemia International Study
ESC: European Society of Cardiology
EAS: European Atherosclerosis Society
hsCRP: high-sensitivity C-reactive protein
HDL-C: high-density lipoprotein cholesterol
LDL-C: low-density lipoprotein cholesterol
LLT: lipid-lowering treatment
MI: myocardial infarction
NSTEMI: non-ST elevation myocardial infarction
SD: standard deviation
STEMI: ST elevation myocardial infarction
TC: total cholesterol
TG: triglycerides

## References

[1] GBD 2013 Mortality and Causes of Death Collaborators (2015). Global, regional, and national age-sex specific all-cause and cause-specific mortality for 240 causes of death, 1990–2013: a systematic analysis for the global burden of Disease Study 2013. Lancet.

[2] Webster MW, Scott RS (1997). What cardiologists need to know about diabetes. Lancet.

[3] Zhou M, Liu J, Hao Y, Liu J, Huo Y, Smith SC (2018). Prevalence and in-hospital outcomes of diabetes among patients with acute coronary syndrome in China: findings from the improving care for Cardiovascular Disease in China-Acute Coronary Syndrome Project. Cardiovasc Diabetol.

[4] Catapano AL, Graham I, De Backer G, Wiklund O, Chapman MJ, Drexel H (2016). 2016 ESC/EAS guidelines for the management of Dyslipidaemias. Eur Heart J.

[5] Malmberg K, Yusuf S, Gerstein HC, Brown J, Zhao F, Hunt D (2000). Impact of diabetes on long-term prognosis in patients with unstable angina and non-Q-wave myocardial infarction: results of the OASIS (Organization to assess strategies for ischemic Syndromes) Registry. Circulation.

[6] Grundy SM, Stone NJ, Bailey AL, Beam C, Birtcher KK, Blumenthal RS, AHA/ACC/AACVPR/AAPA/ABC/ACPM/ADA/AGS/APhA, ASPC/NLA/PCNA Guideline on the Management of Blood Cholesterol. /: A Report of the American College of Cardiology/American Heart Association Task Force on Clinical Practice Guidelines. J Am Coll Cardiol. 2019;73:e285-e350. 10.1016/j.jacc.2018.11.003 30423393

[7] Mach F, Baigent C, Catapano AL, Koskinas KC, Casula M, Badimon L (2020). 2019 ESC/EAS guidelines for the management of dyslipidaemias: lipid modification to reduce cardiovascular risk. Eur Heart J.

[8] Li C, Cui J, Zou L, Zhu L, Wei W (2020). Bioinformatics analysis of the expression of HOXC13 and its role in the prognosis of breast cancer. Oncol Lett.

[9] Zhao S, Wang Y, Mu Y, Yu B, Ye P, Yan X (2014). Prevalence of dyslipidaemia in patients treated with lipid-lowering agents in China: results of the DYSlipidemia International Study (DYSIS). Atherosclerosis.

[10] Ferrières J, Lautsch D, Bramlage P, Horack M, Baxter CA, Ambegaonkar B (2020). Lipid-lowering treatment and low-density lipoprotein cholesterol target achievement in patients with type 2 diabetes and acute coronary syndrome. Arch Cardiovasc Dis.

[11] Gong Y, Li X, Ma X, Yu H, Li Y, Chen J (2021). Lipid goal attainment in post-acute coronary syndrome patients in China: results from the 6-month real-world dyslipidemia international study II. Clin Cardiol.

[12] Ferrières J, Lautsch D, Ambegaonkar BM, De Ferrari GM, Vyas A, Baxter CA (2018). Use of guideline-recommended management in established coronary heart disease in the observational DYSIS II study. Int J Cardiol.

[13] Cosentino F, Grant PJ, Aboyans V, Bailey CJ, Ceriello A, Delgado V (2020). 2019 ESC Guidelines on diabetes, pre-diabetes, and cardiovascular diseases developed in collaboration with the EASD. Eur Heart J.

[14] HPS2-THRIVE Collaborative Group (2013). HPS2-THRIVE randomized placebo-controlled trial in 25 673 high-risk patients of ER niacin/laropiprant: trial design, pre-specified muscle and liver outcomes, and reasons for stopping study treatment. Eur Heart J.

[15] Lee E, Ryan S, Birmingham B, Zalikowski J, March R, Ambrose H (2005). Rosuvastatin pharmacokinetics and pharmacogenetics in white and asian subjects residing in the same environment. Clin Pharmacol Ther.

[16] Zhao SP, Yu BL, Peng DQ, Huo Y (2014). The effect of moderate-dose versus double-dose statins on patients with acute coronary syndrome in China: results of the CHILLAS trial. Atherosclerosis.

[17] Tan NC, Goh CC, Goh SC, Koh YL, Koh KH (2016). The effect of the intensity of lipid-lowering medications on the LDL cholesterol treatment goals of asian patients with dyslipidaemia in primary care. J Clin Pharm Ther.

[18] Fang HSA, Gao Q, Lee ML, Hsu W, Tan NC (2021). LDL-cholesterol change and goal attainment following statin intensity titration among Asians in primary care: a retrospective cohort study. Lipids Health Dis.

[19] Otsuka T, Takada H, Nishiyama Y, Kodani E, Saiki Y, Kato K (2016). Dyslipidemia and the risk of developing hypertension in a Working-Age Male Population. J Am Heart Assoc.

[20] Halperin RO, Sesso HD, Ma J, Buring JE, Stampfer MJ, Gaziano JM (2006). Dyslipidemia and the risk of incident hypertension in men. Hypertension.

[21] Yan X, Li Y, Dong Y, Wu Y, Li J, Bian R (2019). Blood pressure and low-density lipoprotein cholesterol control status in chinese hypertensive dyslipidemia patients during lipid-lowering therapy. Lipids Health Dis.

[22] [Chinese expert consensus on clinical application of selective cholesterol absorption inhibitor. (2015)]. Zhonghua Xin Xue Guan Bing Za Zhi. 2015;43:394-8. 26419982

[23] Masana L, Pedro-Botet J, Civeira F (2015). IMPROVE-IT clinical implications. Should the “high-intensity cholesterol-lowering therapy” strategy replace the “high-intensity statin therapy”?. Atherosclerosis.

[24] Giugliano RP, Cannon CP, Blazing MA, Nicolau JC, Corbalán R, Špinar J (2018). Benefit of adding Ezetimibe to Statin Therapy on Cardiovascular Outcomes and Safety in patients with Versus without Diabetes Mellitus: results from IMPROVE-IT (improved reduction of outcomes: Vytorin Efficacy International Trial). Circulation.

[25] Sabatine MS, Giugliano RP, Keech AC, Honarpour N, Wiviott SD, Murphy SA (2017). Evolocumab and Clinical Outcomes in patients with Cardiovascular Disease. N Engl J Med.

[26] Schwartz GG, Steg PG, Szarek M, Bhatt DL, Bittner VA, Diaz R (2018). Alirocumab and Cardiovascular Outcomes after Acute Coronary Syndrome. N Engl J Med.

[27] Cesaro A, Gragnano F, Fimiani F, Moscarella E, Diana V, Pariggiano I (2020). Impact of PCSK9 inhibitors on the quality of life of patients at high cardiovascular risk. Eur J Prev Cardiol.

[28] Gragnano F, Natale F, Concilio C, Fimiani F, Cesaro A, Sperlongano S (2018). Adherence to proprotein convertase subtilisin/kexin 9 inhibitors in high cardiovascular risk patients: an italian single-center experience. J Cardiovasc Med (Hagerstown).

