# Supplementary material for: Lipid goal attainment in diabetes mellitus patients after acute coronary syndrome: a subanalysis of Dyslipidemia International Study II-China
Source: BMC Cardiovasc Disord. 2023 Jul 1;23:337. doi: 10.1186/s12872-023-03312-w (PMC10315034; doi:10.1186/s12872-023-03312-w)
Supplement: Supplementary file 1 — Additional File Table 1: Demographic, clinical profile and lipid levels at admission of patients included in the final analysis and those discontinued the study [file 12872_2023_3312_MOESM1_ESM.docx]

**Supplementary Table 1.** Demographic, clinical profile and lipid levels at admission of patients included in the final analysis and those discontinued the study

| **Characteristic/category** | **Patients included in the final analysis (*n*=252)** | **Patients discontinued the study (*n*=77)** | ***P*-value** |
| --- | --- | --- | --- |
| **Age**, mean±SD (years) | 63.2±10.5 | 63.8±9.2 | 0.646 |
| **Sex** |  |  | 0.882 |
| Male | 187 (74.2%) | 56 (72.7%) |  |
| Female | 65 (25.8%) | 21 (27.3%) |  |
| **Type of ACS** |  |  | 0.626 |
| STEMI | 66 (26.2%) | 16 (20.8%) |  |
| NSTEMI | 41 (16.3%) | 13 (16.9%) |  |
| UA | 145 (57.5%) | 48 (62.3%) |  |
| **Cigarette smoking status** |  |  | 0.266 |
| Never | 142 (56.3%) | 42 (54.5%) |  |
| Current | 77 (30.6%) | 24 (31.2%) |  |
| Former | 31 (12.3%) | 8 (10.4%) |  |
| Unknown | 2 (0.8%) | 3 (3.9%) |  |
| **DM** |  |  | 0.641 |
| Previously diagnosed | 229 (90.9%) | 72 (93.5%) |  |
| Newly diagnosed | 23 (9.1%) | 5 (6.5%) |  |
| **Sedentary lifestyle** |  |  | 0.206 |
| Yes | 77 (30.6%) | 21 (27.3%) |  |
| No | 147 (58.3%) | 52 (67.5%) |  |
| Unknown | 28 (11.1%) | 4 (5.2%) |  |
| **hsCRP**, median (range), mg/L | 3.8 (0.0-178.0) | 4.8 (0.0-123.8) | 0.972 |
| **Fasting plasma glucose**, mmol/L | 8.6±3.2 | 8.8±3.4 | 0.781 |
| **HbA1c**, % | 7.9±1.6 | 7.6±1.4 | 0.304 |
| **BMI**, mean±SD (kg/m^2^) | 25.5±3.3 | 24.6±3.1 | 0.053 |
| **History of previous ACS** |  |  | 0.841 |
| Yes | 91 (36.1%) | 25 (32.5%) |  |
| No | 146 (57.9%) | 47 (61.0%) |  |
| Unknown | 15 (6.0%) | 5 (6.5%) |  |
| **History of previous MI** |  |  | 0.537 |
| Yes | 44 (17.5%) | 10 (13.0%) |  |
| No | 194 (77.0%) | 61 (79.2%) |  |
| Unknown | 14 (5.6%) | 6 (7.8%) |  |
| **History of ischemic heart disease** |  |  | 0.405 |
| Yes | 46 (18.3%) | 17 (22.1%) |  |
| No | 194 (77.0%) | 54 (70.1%) |  |
| Unknown | 12 (4.8%) | 6 (7.8%) |  |
| **Prior angina** |  |  | 0.079 |
| Yes | 86 (34.1%) | 20 (26.0%) |  |
| No | 151 (59.9%) | 47 (61.0%) |  |
| Unknown | 15 (6.0%) | 10 (13.0%) |  |
| **History of coronary revascularization** |  |  | 0.624 |
| Yes | 63 (25.0%) | 23 (29.9%) |  |
| No | 179 (71.0%) | 52 (67.5%) |  |
| Unknown | 10 (4.0%) | 2 (2.6%) |  |
| **History of TIA** |  |  | 0.129 |
| Yes | 5 (2.0%) | 5 (6.5%) |  |
| No | 231 (91.7%) | 67 (87.0%) |  |
| Unknown | 16 (6.3%) | 5 (6.5%) |  |
| **History of intermittent claudication** |  |  | 0.204 |
| Yes | 1 (0.4%) | 2 (2.6%) |  |
| No | 232 (92.1%) | 69 (89.6%) |  |
| Unknown | 19 (7.5%) | 6 (7.8%) |  |
| **History of peripheral artery revascularization** |  |  | 0.950 |
| Yes | 3 (1.2%) | 1 (1.3%) |  |
| No | 235 (93.3%) | 71 (92.2%) |  |
| Unknown | 14 (5.6%) | 5 (6.5%) |  |
| **History of symptomatic CHF** |  |  | 0.635 |
| Yes | 24 (9.5%) | 6 (7.8%) |  |
| No | 215 (85.3%) | 65 (84.4%) |  |
| Unknown | 13 (5.2%) | 6 (7.8%) |  |
| **Hypertension** |  |  | 0.618 |
| No | 65 (25.8%) | 24 (31.2%) |  |
| Yes, previously diagnosed | 178 (70.6%) | 51 (66.2%) |  |
| Yes, newly diagnosed | 9 (3.6%) | 2 (2.6%) |  |
| **History of CKD** |  |  | 0.489 |
| No | 228 (90.5%) | 67 (87.0%) |  |
| Yes, previously diagnosed | 8 (3.2%) | 5 (6.5%) |  |
| Yes, newly diagnosed | 2 (0.8%) | 0 (0.0%) |  |
| Unknown | 14 (5.6%) | 5 (6.5%) |  |
| **Hypercholesterolemia** |  |  | 0.626 |
| No | 200 (79.4%) | 56 (72.7%) |  |
| Yes, previously diagnosed | 26 (10.3%) | 11 (14.3%) |  |
| Yes, newly diagnosed | 6 (2.4%) | 3 (3.9%) |  |
| Unknown | 20 (7.9%) | 7 (9.1%) |  |
| **History of stroke** |  |  | 0.032 |
| Yes | 20 (7.9%) | 14 (18.2%) |  |
| No | 214 (84.9%) | 57 (74.0%) |  |
| Unknown | 18 (7.1%) | 6 (7.8%) |  |
| **Chronic lung disease** |  |  | 0.846 |
| Yes | 10 (4.0%) | 3 (3.9%) |  |
| No | 228 (90.5%) | 71 (92.2%) |  |
| Unknown | 14 (5.6%) | 3 (3.9%) |  |
| **Lipid levels at admission,** mean±SD (mmol/L) |  |  |  |
| TC | 4.2±1.1 | 4.3±1.3 | 0.619 |
| LDL-C | 2.6±0.9 | 2.6±1.0 | 0.481 |
| HDL-C | 1.0±0.3 | 1.0±0.3 | 0.693 |
| TG | 2.0±1.8 | 1.8±1.3 | 0.485 |
| **LDL-C<1.4mmol/L at admission** |  |  | 0.942 |
| No | 233 (92.5%) | 71 (92.2%) |  |
| Yes | 19 (7.5%) | 6 (7.8%) |  |

SD, standard deviation; ACS, acute coronary syndrome; STEMI, ST elevation myocardial infarction; NSTEMI, non-ST elevation myocardial infarction; UA, unstable angina; DM, Diabetes mellitus; hsCRP, high-sensitivity C-reactive protein; HbA1c: glycated hemoglobin A1c; BMI, body mass index; MI, myocardial infarction; TIA: transient ischemic attack; CHF, chronic heart failure; CKD, chronic kidney disease; TC, total cholesterol; LDL-C, low-density lipoprotein cholesterol; HDL-C, high-density lipoprotein cholesterol; TG, triglycerides.
